# Clinical Usefulness of Bright White Light Therapy for Depressive Symptoms in Cancer Survivors: Results from a Series of Personalized (N-of-1) Trials

**DOI:** 10.3390/healthcare8010010

**Published:** 2019-12-30

**Authors:** Ian M. Kronish, Ying Kuen Cheung, Jacob Julian, Faith Parsons, Jenny Lee, Sunmoo Yoon, Heidis Valdimarsdottir, Paige Green, Jerry Suls, Dawn L. Hershman, Karina W. Davidson

**Affiliations:** 1 Center for Behavioral Cardiovascular Health, Columbia University Irving Medical Center, New York, NY 10032, USA; jej11190@gmail.com (J.J.); fep2110@cumc.columbia.edu (F.P.); sjl2172@cumc.columbia.edu (J.L.); 2 Department of Biostatistics, Mailman School of Public Health, Columbia University Irving Medical Center, New York, NY 10032, USA; yc632@cumc.columbia.edu; 3 Division of General Medicine, Columbia University Irving Medical Center, New York, NY 10032, USA; 4 Department of Oncological Sciences, Icahn School of Medicine at Mount Sinai, New York, NY 10029, USA; heiddisb@ru.is; 5 Department of Psychology, Reykjavik University, 101 Reykjavik, Iceland; 6 Division of Cancer Control and Population Sciences, National Cancer Institute, Rockville, MD 20850, USA; paige.green@nih.gov; 7 Center for Personalized Medicine, Feinstein Institutes for Medical Research, Northwell Health, New York, NY 10022, USA; 8 Department of Medicine, Columbia University Irving Medical Center, New York, NY 10032, USA; dlh23@cumc.columbia.edu

**Keywords:** depression, cancer survivor, bright white light therapy, N-of-1 trials, personalized medicine.

## Abstract

*Purpose*: Little is known about the effectiveness of bright white light therapy (BWL) for depressive symptoms in cancer survivors, many of whom prefer non-pharmacological treatments. The purpose of this study was to compare the effectiveness of BWL versus dim red light therapy (DRL) on depressive symptoms within individual cancer survivors using personalized (N-of-1) trials. *Methods*: Cancer survivors with at least mild depressive symptoms were randomized to one of two treatment sequences consisting of counterbalanced crossover comparisons of three-weeks of lightbox-delivered BWL (intervention) or DRL (sham) for 30 min each morning across 12 weeks. A smartphone application guided cancer survivors through the treatment sequence and facilitated data collection. Cancer survivors tracked end-of-day depressive symptoms (primary outcome) and fatigue using visual analog scales. Within-patient effects of BWL were assessed using an autoregressive model with adjustment for linear time trends. *Results*: Eight of nine cancer survivors completed the 12-week protocol. Two survivors reported significantly (i.e., *p* < 0.05) lower depressive symptoms (−1.3 ± 0.5 and −1.30 ± 0.9 points on a 10-point scale), five reported no difference in depressive symptoms, and one reported higher depressive symptoms (+1.7 ± 0.6 points) with BWL versus DRL. Eight of nine cancer survivors recommended personalized trials of BWL to others. *Conclusions*: There were heterogeneous effects of three-week BWL on self-reported depressive symptoms among cancer survivors, with some finding a benefit but others finding no benefit or even harm. *Implications for Cancer Survivors:* Personalized trials can help cancer survivors learn if BWL is helpful for improving their depressive symptoms.

## 1. Introduction

More than one in five cancer survivors has elevated depressive symptoms, three-fold the prevalence in the general population [1,2,3]. Depressive symptoms are a major contributor to poor quality of life in cancer survivors [4]. Cancer survivors with depressive symptoms are also at risk of poor adherence to preventive therapies, [5,6] worse prognosis, [7,8], and higher health care costs [9]. 

Even though evidence for their effectiveness is often lacking, complementary and alternative medicine (CAM) treatments have become increasingly popular among cancer survivors, with up to 80% of cancer survivors reporting CAM use at least once after diagnosis [10,11,12]. Among CAM treatments for depressive symptoms, there is growing evidence in support of bright white light therapy (BWL) [13]. Recent small randomized clinical trials (RCTs) suggest that BWL may be effective for reducing depressive symptoms and fatigue in cancer survivors [14,15,16]. RCTs, however, estimate the effect of BWL for the average patient in the trial, and provide limited information on the treatment effect that an individual patient can expect to receive.

Personalized trials represent an innovative approach to determining the effectiveness of BWL in cancer survivors [17]. Key features of personalized trials, also known as N-of-1 trials, include a within-subject crossover design with treatment reversals, systematic collection of data on treatment effects, and visualization of data such that patients can share in decision-making about the relative benefits and harms of treatments [18,19]. When conducted successfully, such trials can provide the highest level of evidence for clinical decision-making [20,21]. This design is particularly useful when there is uncertainty regarding whether a treatment is likely to be effective for a given patient [22,23,24]. Thus, personalized trials can help fill the evidence gap regarding BWL by helping cancer survivors learn, through their own guided experiment, whether BWL is helpful for them. 

Accordingly, our goal was to conduct a series of personalized trials comparing BWL with dim red light sham treatment (DRL) within individual depressed cancer survivors. The trials were enabled by a smartphone application that was specifically developed for this use case. We hypothesized that there would be heterogeneous effects of BWL on depressive symptoms, with some patients obtaining significant benefits and others finding no benefits in comparison with DRL.

## 2. Materials and Methods

### 2.1. Customization of N-of-1Trial Smartphone Application and Electronic Platform

We partnered with Overlap Health, Inc. (West Hollywood, Florida) to create a smartphone application for conducting personalized trials of light therapy. The application was designed to (1) guide a patient through a multiple crossover design experiment comparing a bright white (10,000 lux) and a dim red lightbox (50 lux), (2) collect ambulatory assessments of depressive symptoms, fatigue, and side-effects, (3) store and transmit data in a HIPAA-certified manner, (4) aggregate data by treatment period, and (5) visualize data across intervention and sham periods in a manner that helps patients determine whether BWL was effective for them.

### 2.2. Eligibility, Recruitment, and Consent

Implementation of this trial followed the CONSORT (Consolidated Standards of Reporting Trials) 2015 CENT (extension for reporting N-of-1 trials) Statement. (1) Patients were eligible if they were 21 years or older, English speaking, and used an iOS smartphone (i.e., iPhone), a requirement for using the application. Patients also had to have at least mild depressive symptoms (eight-item patient health questionnaire (PHQ-8) score 5–24). (2) Patients who reported suicidal ideations or a history of other severe mental illness (i.e., psychotic disorder or bipolar disorder) were ineligible. Patients were also ineligible if chemotherapy, radiation, or surgery was planned for the subsequent 12 weeks, if life expectancy was less than six months, or if unavailable for follow-up during the 12-week study period.

Patients were identified by promoting the study to clinicians affiliated with Columbia University Irving Medical Center’s (CUIMC) cancer center. The study was also posted on the CUIMC website “Recruit Me”, on Clinicaltrials.gov (NCT0316737), and on various websites and message boards of organizations serving cancer survivors. Flyers included a URL that allowed patients to conduct prescreening electronically. Those who remained eligible after prescreening were invited to meet with the study coordinator to confirm eligibility, to have the full study protocol explained, and to provide written signed informed consent. The Institutional Review Board of CUIMC approved the study protocol (IRB-AAAR1273).

### 2.3. Personalized Trial Protocol

With the assistance of a research coordinator, patients downloaded the smartphone application onto their iPhone from the App Store. Patients were then instructed to use one of two small (6″ × 5″ × 1″), portable lightboxes (Litebook Advantage, Litebook^®^, Ltd. Medicine Hat, Toronto, Canada) each morning for 30 min per day, three weeks at a time, for a total of 12 weeks, or six weeks with each lightbox. They were told to keep the lightbox within their field of vision and within arm’s length during use. One of the lightboxes, labeled A, emitted bright white light (~465 nm) equivalent to an intensity of 10,000 lux when at a distance of 20″–24″ (active treatment). The other lightbox, labeled B, was identical appearing when turned off, but emitted dim red light (~633 nm) at an intensity of 50 lux, an insufficient light intensity to activate the melanopsin-producing cells from the retina (sham treatment) [25]. The application then guided the patient through the personalized trial protocol by sending push notification reminders to use the appropriate lightbox (A or B) each morning and to complete end-of-day assessments of mood and fatigue. At the end of the 12-week period, the patient met with the study coordinator to review the visualizations of their personalized trial data and discuss treatment preferences.

### 2.4. Randomization and Blinding

Randomization to one of two balanced treatment sequences (i.e., bright white-dim red-dim red-bright white or dim red-bright white-bright white-dim red) took place after eligibility was confirmed. Balanced treatment sequences were used to decrease the potential for linear time effects to bias the understanding of treatment effects [19]. A random number generator in the statistical program R was used to assign patients to one of the treatment sequences. The research coordinator then distributed the appropriately labeled lightboxes while being blinded to the treatment sequence.

Patients were informed that the goal of the study was to learn which lightbox was best for them and were not told which lightbox was expected to be the active treatment. Study personnel responsible for assessing outcomes were masked to the treatment sequence assignment until the completion of the personalized trial. The statistician comparing within-patient effects of BWL and DRL was also masked to treatment sequence assignment.

### 2.5. Outcomes

#### 2.5.1. Depressive (Primary) and Fatigue Symptoms

At the end of each day (patients selected the time), patients received a push notification asking them to report their depressive symptoms and fatigue using standard single-item visual analog scale (VAS) items found to be reliable and valid affective symptom measures in prior studies: “How sad or depressed were you today?” (0—not at all depressed to 10—extremely depressed), and “How tired or fatigued were you today?” (0—not at all tired to 10—extremely tired) [26].

#### 2.5.2. Side Effects

During daily push notifications reminding patients to use the appropriate lightbox, patients were also asked whether they had any side-effects from the lightbox, and if so, to describe them using a free text entry.

#### 2.5.3. Lightbox Preference

After completing the personalized trial, patients reviewed the results with the study coordinator, and then stated their preferred treatment (i.e., BWL, DRL, or neither), likelihood of continuing to use the preferred lightbox (1—“not at all likely” to 4—“very likely”), helpfulness of participation in the personalized trial (1—“not at all helpful” to 4—“very helpful”), and how much they would recommend personalized trials of light therapy to other depressed cancer survivors (1—“not at all” to 5—“very much”).

#### 2.5.4. Other Measures

At enrollment, patients’ sociodemographic characteristics (age, gender, race, ethnicity, years of education, employment status, health insurance status), cancer history (type of cancer, treatment modalities), depression history (prior diagnosis of depression, use of antidepressants, psychotherapy, or CAM for depression), other medical history (number of medications and comorbid conditions), and preference for shared decision-making [27] were obtained through self-report. At the final interview, patients were asked to describe the ease of using the smartphone application.

### 2.6. Data Visualization

**Figure A1:**
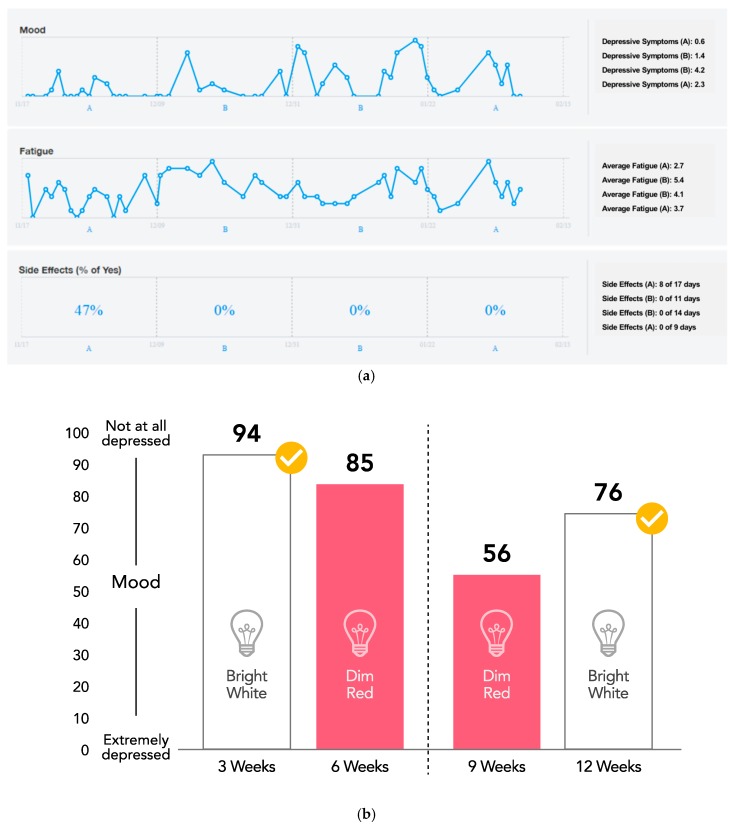
Examples of graphic visualizations of data provided to patients and the study clinician over the course of the personalized trial. (**a**) Real-time visualization of depressive symptoms, fatigue symptoms, and side-effects chronology within the smartphone application. (**b**) Illustrative visualization of average depressive symptom scores by treatment period as shown to patients at the end of the personalized trial. Blue columns referred to symptoms during bright white light treatment periods. Red columns referred to symptoms during dim red light treatment periods. To facilitate interpretation, depressive symptom scores were subtracted from 100 such that higher scores indicated fewer depressive symptoms. The checkmark indicated that differences in lightboxes were statistically significant (i.e., *p* < 0.05 in adjusted model).

Over the course of the personalized trial, patients could view their average symptom scores per treatment period (Figure A1, Panel a). At the end of the trial, they were provided with a column graph that visualized their average symptoms during the personalized trial, grouped by treatment period (Figure A1, Panel b). Statistically significant differences in symptoms were highlighted by including a checkmark corresponding to the lightbox that resulted in lower symptoms.

### 2.7. Sample Size Estimate

In personalized trials, sample size refers to the number of assessments and treatment periods for each patient. As we had no prior data from which to estimate within-patient day-to-day variability in depressive symptoms nor an expected effect size for depressive symptom reduction when comparing BWL to DRL, we did not calculate statistical power a priori for the number of treatment periods or number of assessments per treatment period. Rather, the sample size for each personalized trial was based on collecting the maximal amount of data expected to be tolerable from the perspective of patient study burden. In prior focus groups assessing patient preferences for the design of personalized trials, a 12-week study duration with 5 min of assessments per day was viewed as the upper limit of what patients would comply with, and this corresponded to the duration and intensity of self-assessments used in this study [17].

### 2.8. Statistical Analyses

Descriptive statistics were used to describe patients in the study. To determine whether BWL was superior to DRL for reducing depressive symptoms for individual patients, treatment effects were assessed using an autoregressive model that included the type of light therapy as the main exposure, adjusted for time (e.g., days since enrollment) linearly as a covariate, and accounted for autocorrelations of the order 1. Statistical significance was defined as *p* < 0.05. Given substantial heterogeneity in the within-patient effects of BWL, data were not pooled across patients.

### 2.9. Data Availability

Data available on request from the authors.

## 3. Results

**Figure 1:**
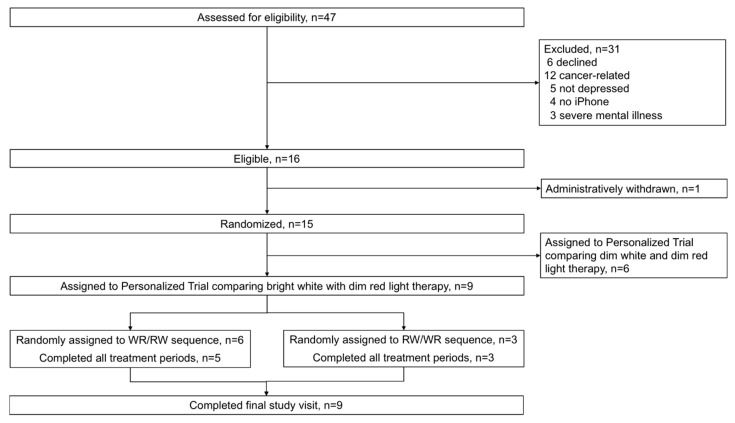
Patient flow diagram. Abbreviations: WR/RW, balanced treatment sequence beginning with bright white light therapy (W, RW/WR, balanced treatment sequence beginning with dim red light (R).

Between 1 September 2017 and 1 May 2018, 47 cancer survivors completed the prescreening questionnaire, and 15 were eligible for the personalized trial of light therapy. Nine cancer survivors were assigned to this series of personalized trials comparing BWL versus DRL (Figure 1). Six patients were assigned to personalized trials comparing two sham treatments (dim white versus dim red light), which are not further discussed here. The mean age of these nine patients was 54 years, 78% were women, 78% were white, and 33% were Hispanic (Table 1). The most common type of cancer was breast cancer (56%). None had metastatic disease. Two-thirds of patients had at least moderate depressive symptoms (i.e., PHQ-8 score ≥ 10) and the mean PHQ-8 score was 10. Most patients (seven out of nine) were already engaging in some form of depression treatment, and most patients (six out of nine) preferred shared decision-making with their doctor.

Overall, eight out of nine patients completed the 12-week personalized trial, the ninth patient dropped out of the trial in the third week due to perceived lack of benefits of either type of light therapy. For the eight patients who completed the protocol, depressive symptom assessments were recorded on 72.0% of possible days (i.e., 60.5 SD 8.8 days out of a possible 84 days total)

### 3.1. Outcomes and Estimation

**Figure 2:**
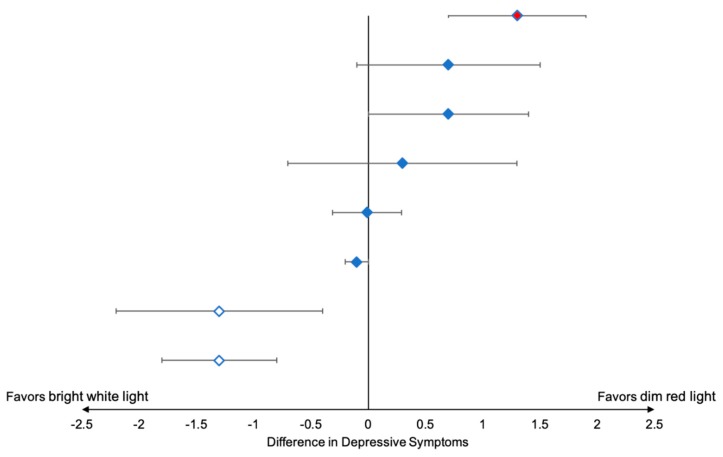
Within-patient differences in depressive symptoms using bright white light therapy (BWL) versus dim red light therapy (DRL). Depressive symptoms were modeled adjusting for linear time trend and accounting for autocorrelations one day apart, *p* < 0.05 denoted statistical significance. White diamond indicates significantly lower depressive symptoms with BWL, red diamond indicates significantly higher depressive symptoms with BWL, and blue diamond indicates no significant difference in depressive symptoms between BWL and DRL.

**Figure A2:**
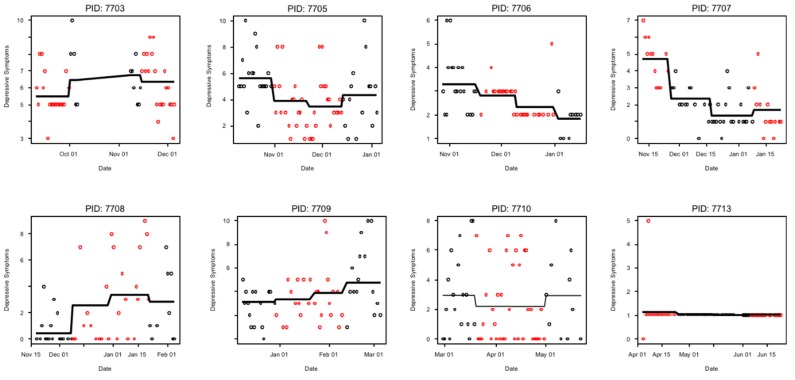
Individual patient-level plots of depressive symptoms over the course of each personalized trial for eight patients who completed the 12-week protocol. Black dots refer to bright white light therapy treatment periods, and red dots refer to dim red light therapy treatment periods. Symptoms were reported at the end of the day using a 0 (no depressive symptoms) to 10 (severe depressive symptoms) visual analog scale.

The results of the individual personalized trials are presented in Table 2 and in Figure A2. Overall, BWL was associated with significantly fewer patient-reported depressive symptoms in two patients, no significant difference in five patients, and worse depressive symptoms in one patient (Figure 2). A similar pattern was observed with respect to fatigue symptoms, with just one patient reporting lower fatigue, six patients reporting no difference in fatigue severity, and one patient reporting higher fatigue with BWL versus DRL.

### 3.2. Harms

Only one patient reported any adverse effects from either lightbox. This patient reported mild headaches during eight days of the first three-week treatment period with BWL. However, these headaches were not severe enough for the patient to discontinue the personalized trial, and this side-effect was not reported during the second BWL treatment period.

### 3.3. Light Therapy Preferences and Overall Satisfaction with Personalized Trial

Overall, four patients preferred BWL, one preferred DRL, and four had no preference. Five patients reported being likely to continue using their preferred lightbox after the personalized trial was over. The patient who preferred DRL reported that he found the light soothing and helpful with his morning meditation. Preferences for light therapy were consistent with the data presented to patients in seven out of nine patients, two patients preferred BWL despite reporting no significant differences in depressive symptoms between lightboxes. With respect to overall satisfaction, six patients would “very much” recommend and two would “a little bit” recommend participation in a personalized trial of light therapy to other cancer survivors with depressive symptoms, only one patient would not recommend participation. All patients reported that the application was easy to use, and none were bothered by the effort needed to self-track mood and fatigue. Those few concerns that were raised regarding personalized trial participation pertained to the time burden and inconvenience of completing 30 min of light therapy each morning.

## 4. Discussion

This study addressed whether smartphone application-delivered personalized trials could be a helpful tool for personalizing the selection of BWL for depressive symptoms in cancer survivors. Substantial heterogeneity in the effect of BWL on depressive symptoms was uncovered, with a subset of patients finding benefits, a subset finding no benefit, and, in one patient, possibly harm. While a small sample, two out of nine patients identified a clinically significant reduction in self-reported depressive symptoms over a sham, which is comparable to the number needed to treat with antidepressants for one patient to obtain benefits over placebo in primary care [28]. Of note, the effectiveness of antidepressant medications in cancer survivors remains uncertain [29].

One surprising finding was that one patient reported higher depressive symptoms with BWL compared to DRL, and this patient preferred DRL over BWL. To our knowledge, prior studies have not reported depressogenic effects of BWL. Future studies should seek to determine if this finding is replicable, and if so, through which biological mechanisms. One possibility is that rather than BWL causing harm, DRL could have had a beneficial effect. DRL at the intensity provided in the study was expected to be physiologically inert with respect to the melanopsin pathway [30]. Nevertheless, there could have been other pathways through which DRL exerted its effects. The one patient who preferred DRL believed that it induced a calming effect. This anecdote highlights the unique potential for personalized trials to provide patients with singular insights that could never be appreciated in a conventional RCT in which participants are only exposed to one treatment. Future studies of light therapy should seek to better understand the heterogeneous effects of BWL and DRL. Pairing personalized trials with biological data (e.g., melatonin levels or 24-hour rest-activity behavioral rhythms) to more deeply phenotype patients may help better understand the effects of light therapy and could result in biomarkers that personalize the selection of light therapy without necessitating an N-of-1 experimental design.

Few prior studies have employed a personalized trials approach to individualize the selection of treatments for depression, and to our knowledge, none have evaluated the effectiveness of light therapy in this manner [31]. One of the requirements of the multiple crossover design component of personalized trials is that treatments are reversible and have a sufficiently short onset and washout period to enable crossovers to occur in a feasible time period. Psychotherapy is often considered insight-oriented, and so is not easily reversible. While conventional antidepressant medications are theoretically reversible, and so technically amenable to a personalized trial approach, withdrawal effects and the timeline for onset and offset suggest that other treatments, such as light therapy, may be more suitable for testing with multiple crossover designs.

### 4.1. Limitations

There were several limitations to our study. First, we assessed depressive symptoms using a single end-of-day VAS question, and this is not analogous to understanding treatment effects using a psychiatric interview or other more detailed depressive symptom assessments. Nevertheless, single item mood scales are more feasible for daily administration and have been strongly associated with longer instruments and clinical interviews in prior studies [26,32]. Our protocol included only one treatment repetition and our analysis plan accounted for only two possible confounders (i.e., linear trend, autocorrelation), and thus may have misattributed differences in depressive symptoms to BWL. The treatment period was limited to three weeks. While prior studies have shown that BWL can reduce depressive symptoms in as few as two weeks, a longer duration of BWL may have been needed to impact depressive symptoms in some patients [33]. The impact of seasonality was not considered. Adherence to light therapy was not objectively assessed. Future personalized trials of this nature may wish to better establish baseline symptoms prior to initiating light therapy to help clarify treatment effects. A small number of patients were enrolled. Thus, assessments of the feasibility of the smartphone-delivered personalized trial and of the pooled effect of BWL on depressive symptoms should be interpreted cautiously.

### 4.2. Generalizability

We enrolled a small number of English-speaking cancer survivors who use smartphones into our study, which limits the understanding of the generalizability of the findings to other cancer survivors. Nevertheless, smartphones are increasingly ubiquitous, and an application could easily be translated into Spanish [34]. We also excluded cancer survivors undergoing primary cancer treatments, as personalized trials are challenging to interpret when multiple possible confounders of treatment effects occur across time.

## 5. Conclusions

The personalized trials approach used in this study brought back the focus on the individual person that existed before the convention of pooling across many persons in between-subject RCTs [35]. We determined that there were heterogeneous treatment effects for BWL, with some patients finding a benefit and others no benefit or even harm. Further, the majority of patients recommended personalized trials of light therapy to others. Personalized trials may be ideally suited to testing the effectiveness of relatively short-acting depression treatments in cancer survivors, particularly emerging treatments for which we have a limited understanding of treatment effects.

## Figures and Tables

**Table 1:** Baseline patient characteristics (N = 9).

Characteristic	Mean (SD) or *n* (Percentage)
Age in years, mean (SD)	54.2 (17.7)
Female	7 (78%)
Hispanic	3 (33%)
Race:	
White	7 (78%)
Black	1 (11%)
Other	1 (11%)
Cancer type:	
Breast	5 (56%)
Thyroid	2 (22%)
Bone	1 (11%)
Skin	1 (11%)
Prior cancer treatment:	
Surgery	7 (78%)
Chemotherapy	4 (44%)
Radiation	3 (33%)
Hormonal	4 (44%)
Depressed (PHQ8 ≥ 10)	6 (67%)
PHQ-8 score, mean (SD)	9.7 (2.2)
Current depression treatment:	
Receiving any depression treatment	7 (78%)
Prescribed antidepressants	4 (44%)
Receiving psychotherapy	6 (67%)
Complementary and alternative medicine	3 (33%)
Decision-making preference:	
I want doctor to decide	0 (0%)
I want doctor to consider my ideas	1 (11%)
I want doctor and I to make decisions together	6 (67%)
I want to decide	2 (22%)

PHQ-8—patient health questionnaire; SD—standard deviation.

**Table 2:** Characteristics and outcomes of personalized trials of light therapy for depressive symptoms (N = 9).

Patient	Season (Months)	Treatment Sequence	Percent of Days with Symptom Assessments	Mean Depressive Symptoms (0, None to 10, Severe)	Mean Fatigue Symptoms (0, None to 10, Severe)	Treatment Preference at end of Trial	Likelihood of Continuing Light Therapy (Not at All to Very Much)	Helpfulness of Participation (Not at All to Very Much)	Recommend to Others (Not at All to Very Much)
1 (7702)	Fall (September-December)	W-R-R-W	19%	Insufficient	Insufficient	Neither	Not at all likely to continue	Not at all helpful	Recommend a little bit
2 (7703)	Fall (September-December)	R-W-W-R	62% ^†^	R: 5.9 W: 6.7 Difference: 0.7 SE 0.8, *p* = 0.40	R: 7.0 W: 8.2 Difference: 1.3 SE 0.5, *p* = 0.001	R	Somewhat likely to continue	Somewhat helpful	Recommend a little bit
3 (7705)	Fall (October–January)	W-R-R-W	80%	R: 3.6 W: 5.1 Difference: 1.3 SE 0.6, *p* = 0.04	R: 4.7 W: 5.7 Difference: 0.9 SE 0.7, *p* = 0.17	R	Somewhat likely to continue	Very much helpful	Recommend strongly
4 (7706)	Fall (November–January)	W-R-R-W	64%	R: 2.6 W: 2.8 Difference: −0.01 SE 0.3, *p* = 0.98	R: 2.6 W: 2.6 Difference: −0.2 SE 0.2, *p* = 0.43	Neither	Not at all likely to continue	Not at all helpful	Recommend not at all
5 7707	Fall (November–January)	R-W-W-R	66%	R: 3.0 W: 1.8 Difference: −1.3 SE 0.5, *p* = 0.005	R: 3.8 W: 3.7 Difference: −0.3 SE 0.5, *p* = 0.59	W	Very likely to continue	Very much helpful	Recommend strongly
6 7708	Winter (November–February)	W-R-R-W	57%	R: 3.0 W: 1.3 Difference: −1.3 SE 0.9, *p* = 0.17	R: 4.6 W: 2.9 Difference: −1.7 SE 0.7, *p* = 0.03	W	Very likely to continue	Very much helpful	Recommend strongly
7 7709	Winter (December–March)	W-R-R-W	64%	R: 3.6 W: 4.0 Difference: 0.3 SE 1.0, *p* = 0.74	R: 4.8 W: 6.0 Difference: 0.6 SE 1.0, *p* = 0.55	W	Very likely to continue	Very much helpful	Recommend strongly
8 7710	Spring (March–June)	W-R-R-W	77%	R: 2.2 W: 3.0 Difference: 0.7 SE 0.7, *p* = 0.32	R: 3.1 W: 3.4 Difference: 0.3 SE 0.6, *p* = 0.60	Either	Unlikely to continue	Somewhat helpful	Recommend strongly
9 7713	Spring (April–June)	R-W-W-R	89%	R: 1.1 W: 1.0 Difference: −0.1 SE 0.1, *p* = 0.51	R: 7.3 W: 7.1 Difference: −0.2 SE 0.4, *p* = 0.65	Either	Unlikely to continue	A little bit helpful	Recommend strongly

Abbreviations: W, bright white light therapy; R, dim red light therapy; SE, standard error. Depressive and fatigue symptoms were assessed at the end of each day on a scale from 0 (none) to 10 (severe). ^†^ Assessments were interrupted in the middle third of the study due to an interruption in the smartphone application.
